# Subcellular partitioning of protein kinase activity revealed by functional kinome profiling

**DOI:** 10.1038/s41598-022-21026-5

**Published:** 2022-10-15

**Authors:** Lauren Wegman-Points, Khaled Alganem, Ali Sajid Imami, Victoria Mathis, Justin Fortune Creeden, Robert McCullumsmith, Li-Lian Yuan

**Affiliations:** 1 Department of Physiology and Pharmacology, College of Osteopathic Medicine, Des Moines University, Des Moines, IA 50312 USA; 2 Department of Neurosciences, College of Medicine and Life Sciences, University of Toledo, Toledo, OH 43614 USA; 3 Neurosciences Institute, ProMedica, Toledo, OH USA

**Keywords:** Biological techniques, Neuroscience

## Abstract

Protein kinases and their substrates form signaling networks partitioned across subcellular compartments to facilitate critical biological processes. While the subcellular roles of many individual kinases have been elucidated, a comprehensive assessment of the synaptic subkinome is lacking. Further, most studies of kinases focus on transcript, protein, and/or phospho-protein expression levels, providing an indirect measure of protein kinase activity. Prior work suggests that gene expression levels are not a good predictor of protein function. Thus, we assessed global serine/threonine protein kinase activity profiles in synaptosomal, nuclear, and cytosolic fractions from rat frontal cortex homogenate using peptide arrays. Comparisons made between fractions demonstrated differences in overall protein kinase activity. Upstream kinase analysis revealed a list of cognate kinases that were enriched in the synaptosomal fraction compared to the nuclear fraction. We identified many kinases in the synaptic fraction previously implicated in this compartment, while also identifying other kinases with little or no evidence for synaptic localization. Our results show the feasibility of assessing subcellular fractions with peptide activity arrays, as well as suggesting compartment specific activity profiles associated with established and novel kinases.

## Introduction

Intracellular structure and organization are highly dynamic and heterogenous. Cellular function requires the precise temporal and spatial choreography of its components for functions from cellular metabolism to intercellular signaling^1^. At a basic organizational level, most cells exhibit polarity, manifested as differences in structure and function from one end of the cell to another. This organizational tenet allows precise control over the transportation (e.g. secretion or absorption) of substrates across cellular compartments. Additionally, membranous organelles localize cellular functions and biochemical reactions to within well-defined boundaries. Proteases are largely confined to lysosomes to precisely regulate the turnover of proteins. The nucleus contains cohorts of proteins involved in the regulation and execution of transcriptional programs. These principles are conserved from simple epithelial cells to highly complex cortical neurons. Neurons, with their elaborate architecture and role in input integration, require additional functional compartments. Dendritic spines, the postsynaptic sites of most excitatory input, allow for the functional isolation of each postsynaptic site from its neighboring input. The molecular segregation of the spine from the dendritic shaft allows for the communication between each spine and its associated presynaptic terminal to be strengthened or weakened independently^2^. Modification of each synapse is therefore dependent on its own activity and not that of neighboring synapses.

Throughout the cell, the most common post-translational protein modification used in signaling complexes is phosphorylation, carried out by the superfamily of protein kinases^3^. Many kinases have more than one molecular target within a cell, making subcellular localization critical to their function. In some cases, the localization and/or compartmentalization of certain kinases can be deduced by their function. Extracellular signal-related kinase (ERK) shuttles between the cytosol and nucleus to perform its function directing cell proliferation and differentiation in response to extracellular signaling^4^. However, there are other kinases whose localization and activity relationship may not be so intrinsically apparent. If little is known about a particular kinase’s function, determining its spatial profile can be a significant step towards determining its function within a particular cell type. There are many examples of the subcellular localization of kinase activity in neurons where their trafficking is critical to their function. For instance, increased activity of the kinase for eukaryotic elongation factor 2 (eEF2K) in dendritic spines is critical for local protein translation and synaptic strengthening downstream of increased glutamate signaling^5^.

There are a number of biochemical assays available to explore kinase expression and function within a cell. Immunohistochemistry and Western blot can provide some information on the localization and expression level of specific kinases. These conventional biochemical assays require known targets with kinase-specific components (e.g. antibodies) and are limited by their inability to study more than a single or a small number of kinases at once^6^. Kinome array profiling allows the measurement of the activity of many kinases at once, but historically lacks spatial resolution. Here we combine the power of kinome array profiling with spatial resolution to generate a refined look at the signaling pathways within subcellular compartments.

The PamSation12 platform (PamGene International) is a well-established microarray technology for multiplex kinase activity profiling^7–9^. The array chips are composed of a grid of reporter peptides that can be phosphorylated by active kinases present in the sample, which are then detected by a fluorescent antibody. The 3-D structure of the chips facilitates binding and subsequent enzymatic reactions, resulting in extremely high sensitivity. Additionally, the platform allows for high- throughput assessment of kinase activity which provides a rich data set to model kinase interaction networks. This feature can be used to model the underlying biology of how kinase networks interact and function which is a key component to study kinases holistically^10^. To date there have not been studies using this technology to examine the differences in kinase activity between various cellular compartments. Furthermore, restricting experiments to the cytosolic fraction risks the loss of critical kinases located in discrete cellular compartments, such as the nucleus and the synapse.

Here we deploy an unbiased kinome array profiling approach to assess cytosolic, synaptosomal, and nuclear subcellular fractions. As a proof of concept, we utilized arrays hosting Ser/Thr kinase (STK) targets. By examining kinase activity between nuclear, cytosolic, and synaptosomal compartments, we begin to provide an in-depth analysis of the subcellular compartmentalization of kinase activity. Here we present evidence of specific subcellular kinome activity, with a particular focus on the synapse.

## Materials and methods

### Animals

Male Sprague-Dawley rats were purchased from Charles River (Wilmington, MA) at the weight of 165–200 g. They were paired housed and kept under a 12 h light/dark cycle at constant temperature (25 °C) and humidity with ad lib access to food and water. Animals were acclimated to our husbandry facility for at least one week prior to the start of experimentation. All the experiments were carried out in accordance with the National Institutes of Health guide for the care and use of laboratory animals. The Animal Care and Use Committee of the Des Moines University approved this study. The study is reported in accordance with ARRIVE guidelines.

### Cellular fractionation of rat cortical tissue

Rats were sacrificed by guillotine and brain tissue was collected on ice and kept at − 80 °C until processing. The cortical tissue was homogenized in a buffer containing 0.32 M sucrose, 20 mM HEPES, 1 mM EDTA, 5 mM NaF, 1 mM NaVO_3_, protease inhibitor cocktail and phosphatase inhibitor cocktail. Centrifugation of the tissue homogenate at 2.8 krpm for 10 min resulted in a pellet (P1: nuclear fraction). The supernatant was transferred to a clean microtube and spun down at 12 krmp, resulting in a second pellet (P2: synaptoneurosomal fraction) and a supernatant (cytosolic fraction). Pellets were resuspended in RIPA buffer. Tissue protein concentrations were determined using a Bicinchoninic Acid Assay (BCA) kit (Pierce; Rockford, IL).

### Western blotting

Equal amounts of protein (20 µg) from the three fractions were loaded into a 10% polyacrylamide gel and transferred to nitrocellulose membranes. Membranes were blocked in 5% bovine serum albumin (BSA) in Tris-buffered saline containing 0.1% Tween 20 (TBST). Membranes were then incubated overnight at 4 °C with primary antibodies (anti-GluR1, 1:1,000, Millipore,; anti-PSD95, 1:5,000, NeuroMab; anti-b-actin, 1:400,000, Cell Signaling technology; anti-GAPDH, 1:1,000, Proteintech; anti- acetyl-Histone H3, 1:20,000, Millipore). After multiple washes with TBST, membranes were incubated with fluorescence-conjugated secondary antibody for 2 h at room temperature. All samples were run simultaneously. The membrane was imaged using the Odyssey imaging system (LI-COR) and its associated software. No bands were quantified as we used this Western blotting to show qualitative differences between cellular fractions. Full gels of western blot with molecular weight markers are shown in Supplementary Fig. S1.

### Kinome array profiling of Ser/Thr kinase activity

The kinome activity profiling platform, PamStation12, was used to measure serine/threonine kinome activity using the STK PamChip (PamGene International).

Each PamChip has 4 wells allowing 4 samples to be loaded on a single chip and the Pamstation12 can accommodate 3 chips per run. An array located in each well on the STK PamChip contains 144 serine/threonine phosphopeptide sequences that are immobilized on a porous membrane. The assay is based on the PamStation12 standard protocols and was performed as described previously^11^. Briefly, three fractions samples (nuclear, cytosolic, and synaptosomal) along with unfractionated brain homogenate were diluted to 0.2 µg/µL loaded on a single chip and ran in triplicate using 3 separate STK PamChips. Identical protein amounts were loaded for each condition. The assay performed by blocking each array with 2% bovine serum albumin (BSA) before 2 ug of protein of the samples, and 157 µM adenosine triphosphate (ATP), and a primary antibody mixture as a part of the two-step reaction process designed for STK PamChips. For the second step, FITC-labeled anti-phospho serine-threonine antibodies (PamGene) were added to each array. The homogenized samples alongside the assay mix were pumped through the wells through timed cycles to catalyze the reaction between kinases in the sample and the reporter peptides on the chip. The degree of phosphorylation per well was measured in real time using Evolve (PamGene) kinetic image capture software. The Evolve software captures images of FITC-labeled anti-phospho antibodies binding to each phosphorylated peptide substrate for each timed cycle. Peptide spot intensity was captured across multiple exposure times (10, 20, 50, 100, 200 ms) during the post-wash phase. The BioNavigator software (PamGene) was used to convert the captured images to numerical values based on the intensity levels. Additional details for the kinome array workflow are included in the supplementary methods.

### Upstream kinase analysis

To identify upstream kinases based on differences in phosphorylation of reporter peptides on the STK chip, three different software packages were used: Kinome Random Sampling Analyzer (KRSA), upstream kinase analysis (UKA), and Kinase Enrichment Analysis (KEA3). To look at associated upstream kinase families, KRSA takes the list of differentially phosphorylated peptides and uses a random resampling approach to assign scores for each kinase family^12^. Additionally, the Upstream Kinase Analysis (UKA) tool from BioNavigator was used to look at individual upstream kinases. The default settings of the standard STK analysis protocol were used with the additional step of upstream kinase analysis. UKA reports the final score as a metric for ranking implicated kinases. The kinase final score is calculated based on the specificity of the peptides mapped to the kinases and the significance of phosphorylation changes of the peptides. The Kinase Enrichment Analysis Version 3 (KEA3) web tool was also used to perform kinase set enrichment analysis using the corresponding proteins of the top differentially phosphorylated reporter peptides as the input. More details for these packages are provided in the supplementary methods section.

We used the Creedenzymatic R package to aggregate the results from these three different analytic tools. The Creedenzymatic R package is a pipeline software package that combines, scores, and visualizes the results from multiple upstream kinase analytic tools (https://github.com/CogDisResLab/creedenzymatic). The Creedenzymatic package harmonizes the different metrics used in KRSA, UKA, and KEA3 with percentile rank normalization. This harmonization results in a unified percentile score for each kinase under each tool. Then, the mean and median percentile score for each kinase is calculated by averaging the normalized scores across the three analytic tools. Additionally, kinases are mapped to the official HUGO Gene Nomenclature Committee (HGNC) symbols and subfamilies, ensuring the naming convention is consistent across the three different tools^13^. More details for this package are provided in the supplementary methods section.

### Gene set enrichment analysis

Two online tools were used to perform gene set enrichment analysis, SynGO and Enrichr. SynGO is a highly curated knowledge base database annotating synapse protein locations and functions^14^. The Enrichr web tool was used to perform multiple gene set enrichment analyses across different get set libraries^15^. To expand the gene set for the enrichment analysis, the STRING database was used to extract the top 20 associated proteins for each enriched kinase (minimum required interaction score was set as 0.5)^16^.

## Results

### Overall workflow

**Figure 1:**
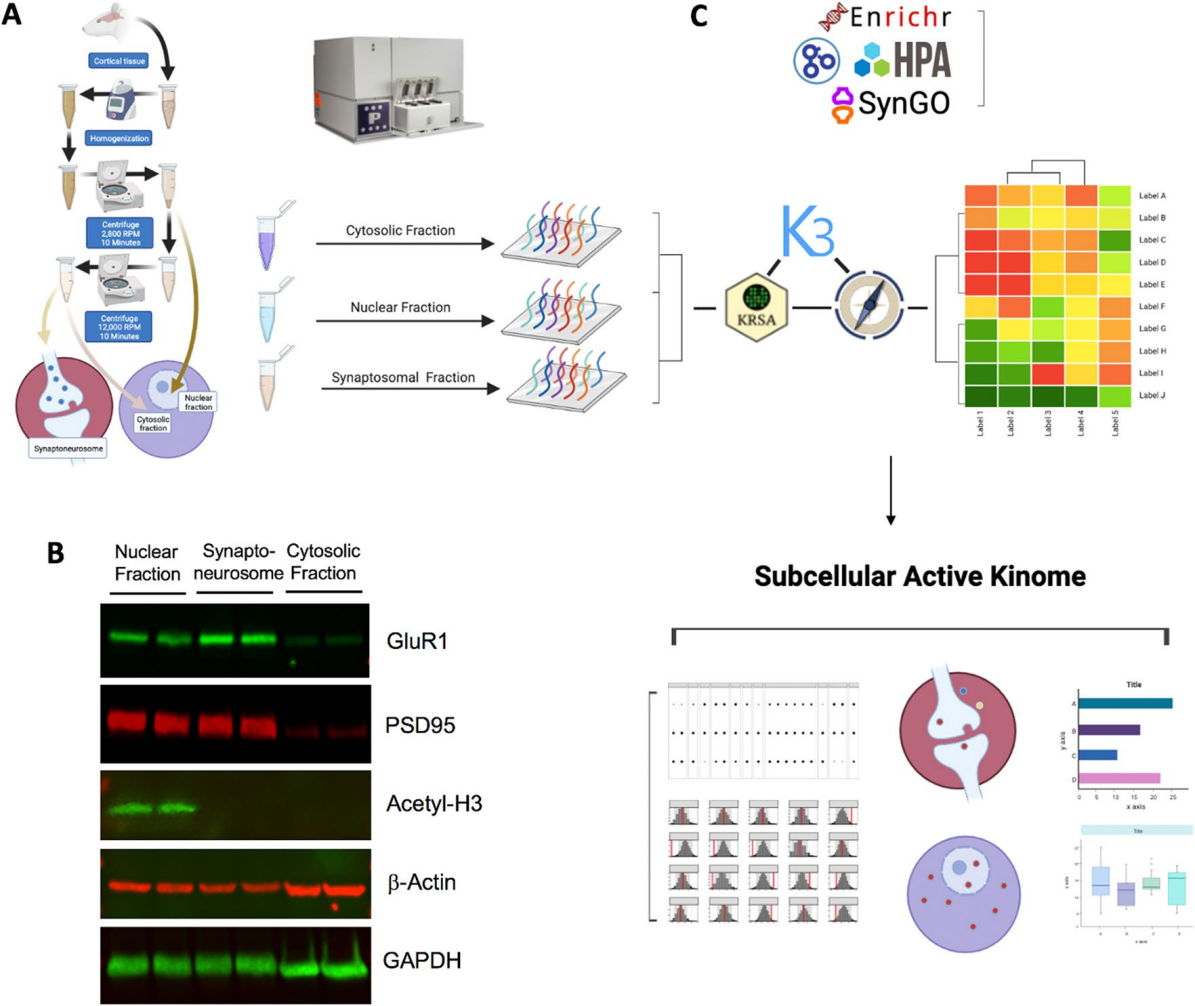
Subcellular Active Kinome Profiling Workflow. (**A**) General steps to isolate nuclear, cytosolic, and synaptoneurosome fractions from rat cortical tissue, as described in Materials and Methods. (**B**) To confirm the subcellular enrichment, three fractions were examined by Western blotting for levels of synaptic proteins GluR1 and PSD95, a nuclear protein acetyl Histone3, and proteins that are evenly distributed across compartments, b-actin and GAPDH. (**C**) Fractionated samples are profiled using the PamStation12 platform for high- assessment of kinase activity. The Serine/Threonine PamChip4 reporter peptides were clustered using unsupervised hierarchical clustering to identify differences between the samples. Three software packages were used to analyze the kinome array data and identify differentially active upstream kinases; KRSA, KEA3, and UKA. Other publicly available tools and databases (Enrichr, HPA, SynGo, GO) are used to perform gene set, pathway, and protein localization enrichment analysis. KRSA: Kinome Random Sampling Analyzer, KEA3: Kinase Enrichment Analysis, UKA: Upstream Kinase Analysis, HPA: Human Protein Atlas, GO: Gene Ontology.

As illustrated in Fig. 1, the overall workflow for the subcellular active kinome profiling process includes (A) protein extraction from subcellular fractions and kinome array profiling, (B) confirmation of subcellular enrichment, and (C) software packages for bioinformatical analysis. Nuclear, cytosolic, and synaptosomal fractions along with unfractionated brain homogenate were pooled from three biological samples, loaded on a single chip, and ran in triplicate using 3 separate STK PamChips. Data were analyzed within a chip, and log2 fold change data was averaged across technical replicates.

### Comparison of Serine/Threonine kinome across three fractions

**Figure 2:**
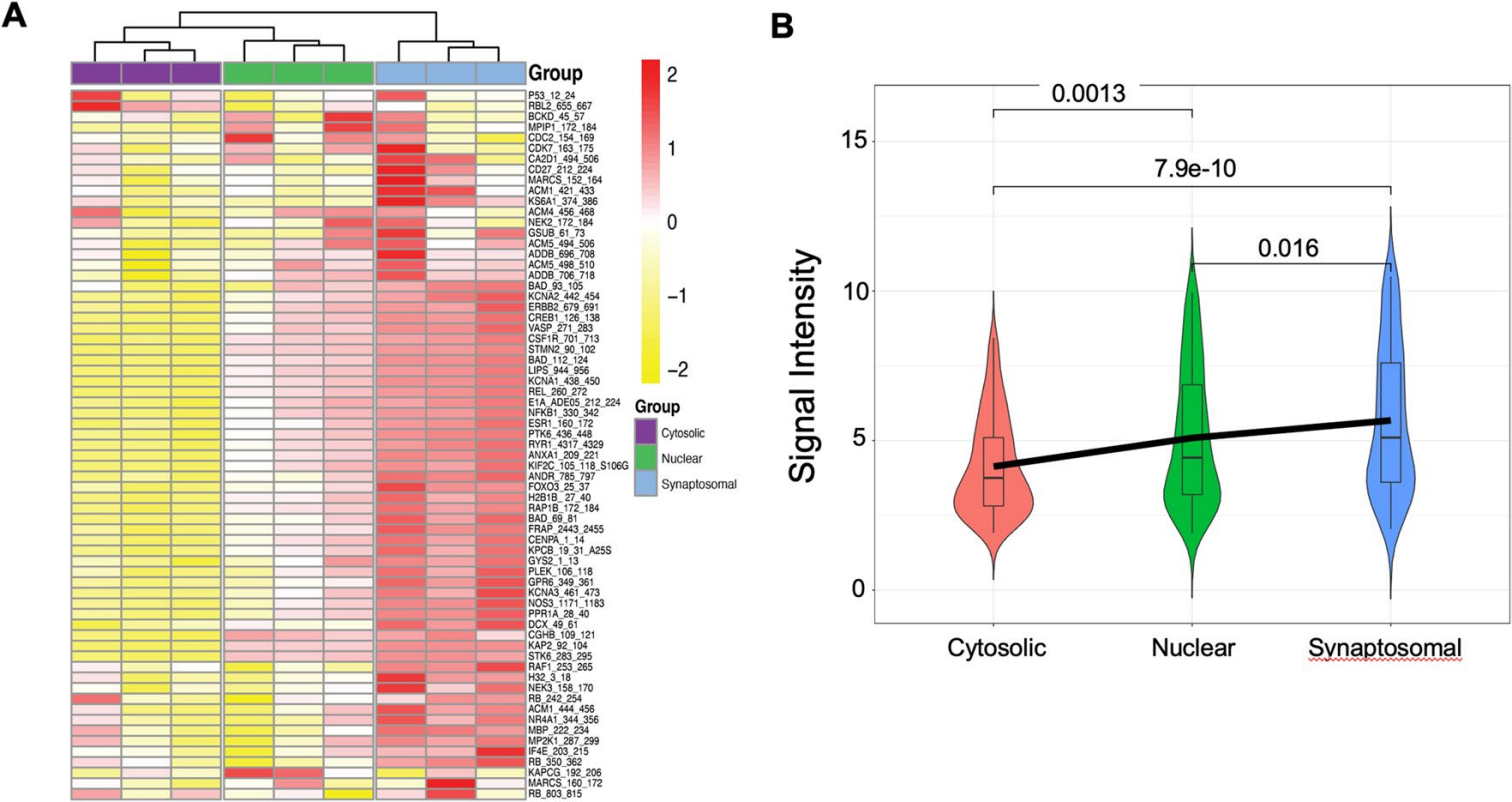
Global Phosphorylation Signals Show Distinct Patterns for Cytosolic, Nuclear and Synaptosomal Fractions. (**A**) Unsupervised hierarchical clustering of the endpoint signal intensity for all of the STK PamChp4 reporter peptides that passed quality control. The values are normalized by peptide (Z-transformed) to highlight the differences between the samples. (**B**) Violin Plots showing the mean global intensity for each group. Statistical comparisons were performed using Wilcoxon’s rank sum test for nuclear vs cytosolic (*p* < 0.001), synaptosomal vs cytosolic (*p* < 0.001) and synaptosomal vs nuclear *p* = 0.016) fractions. The heatmaps and violin plots were generated using KRSA package (v0.10.3—https://github.com/CogDisResLab/KRSA) using R version 4.1.2.

To determine the global phosphorylation signals in the different subcellular fractions, we used KRSA to generate global heatmaps with unsupervised hierarchical clustering using a set of peptides that passed quality control steps. The quality control steps include filtering out peptides that very low signals (< 2 in the 200 ms exposure time) or non-linear slopes. To better highlight the differences between samples, the values are scaled by peptide (Z score). The clustering shows distinct kinome profiles for each subcellular fraction (Fig. 2A). KRSA was also used to generate boxplots representing the global phosphorylation signals in each subcellular fraction while highlighting the average signals intensity. Using equal amount of protein loaded for each sample (2ug), the global signals indicate a relatively higher phosphorylation signals in the nuclear and synaptosomal fractions compared to the cytosolic fraction (Fig. 2B).

To assess variability across chips, we calculated the coefficient of variability (CV) across the three replicate chips for each peptide. These data indicate the median CV values across all three chips for each group are < 9% which is the manufacturer standard for acceptable variability. Cytosolic CV = 4.67%, synaptosomes = 3.91%, and nuclear = 5.69%, total (unfractionated brain homogenate) = 4.55% (Fig. S2).

### Kinome comparison between the synaptosomal and nuclear fractions

**Figure 3:**
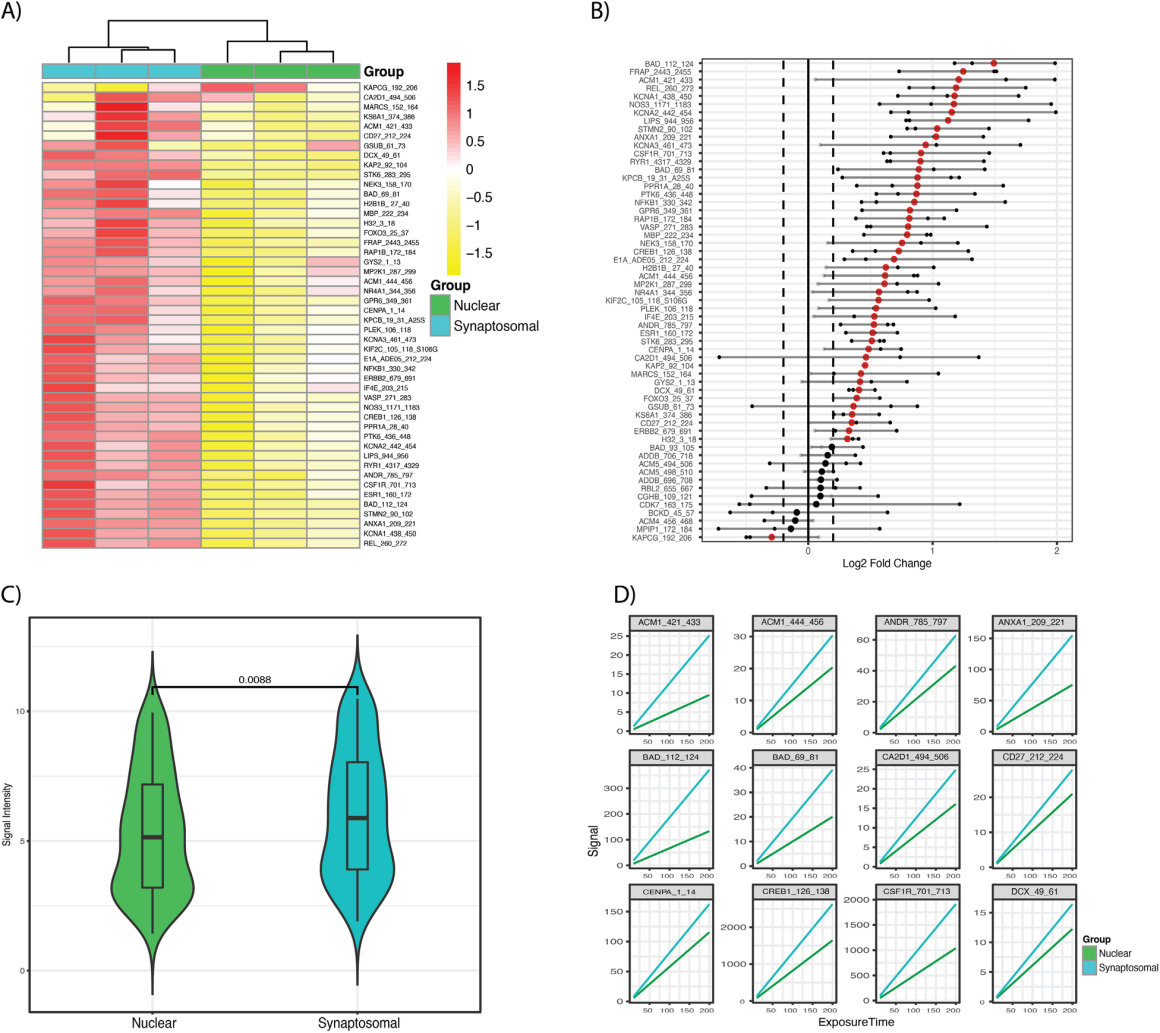
Active Kinome Profiles Comparison of Synaptosomal versus Nuclear Fractions. Two-way comparison of the synaptosomal versus nuclear fractions (nuclear is treated as the “control”). (**A**) Unsupervised hierarchical clustering of the signal intensity for the top differentially phosphorylated peptides. The values are normalized by peptide (Z-transformed) and represent relative phosphorylation. The heatmaps were generated using KRSA package (v0.10.3—https://github.com/CogDisResLab/KRSA) using R version 4.1.2. (**B**) Waterfall showing the Log2 fold change of signal intensity across three chips (smaller points) and the mean Log2 fold change (large point). The color indicates if the mean Log2 fold change is larger than 0.2 or smaller than − 0.2. (**C**) Boxplot showing the mean global signal intensity of the two groups (synaptosomal and nuclear fractions) with a Wilcoxon signed-rank test. (**D**) The signal intensity as a function of exposure time (milliseconds) for a subset of the differentially phosphorylated peptides.

To determine the differences between synaptosomal and nuclear fractions, log2 changes (Log2FC) of signal intensities between the sample groups for each peptide were calculated using only these two datasets for the analyses. A log2FC threshold cutoff (+ /− 0.15) was set to represent meaningful changes in the degree of phosphorylation of peptides between the compared groups. This threshold was chosen based on previous reports suggesting that this magnitude of change in phosphorylation or kinase activity reflects significant biological consequences^17–19^. 48 peptides had differential phosphorylation between synaptosomal and nuclear fractions with distinct phosphorylation signatures (Fig. 3A). 47 out of the 48 peptides showed increased phosphorylation in the synaptosomal fractions (Fig. 3B). The results indicate a significant difference between synaptosomal and nuclear fractions (*p*-value = 0.0088 − Wilcoxon signed-rank test) (Fig. 3C). The signal intensity as a function of exposure time (milliseconds) showed a higher phosphorylation signal in the synaptosomal fraction compared to the nuclear fraction (Fig. 3D).

### Identification of upstream kinases with enriched activity

**Figure 4:**
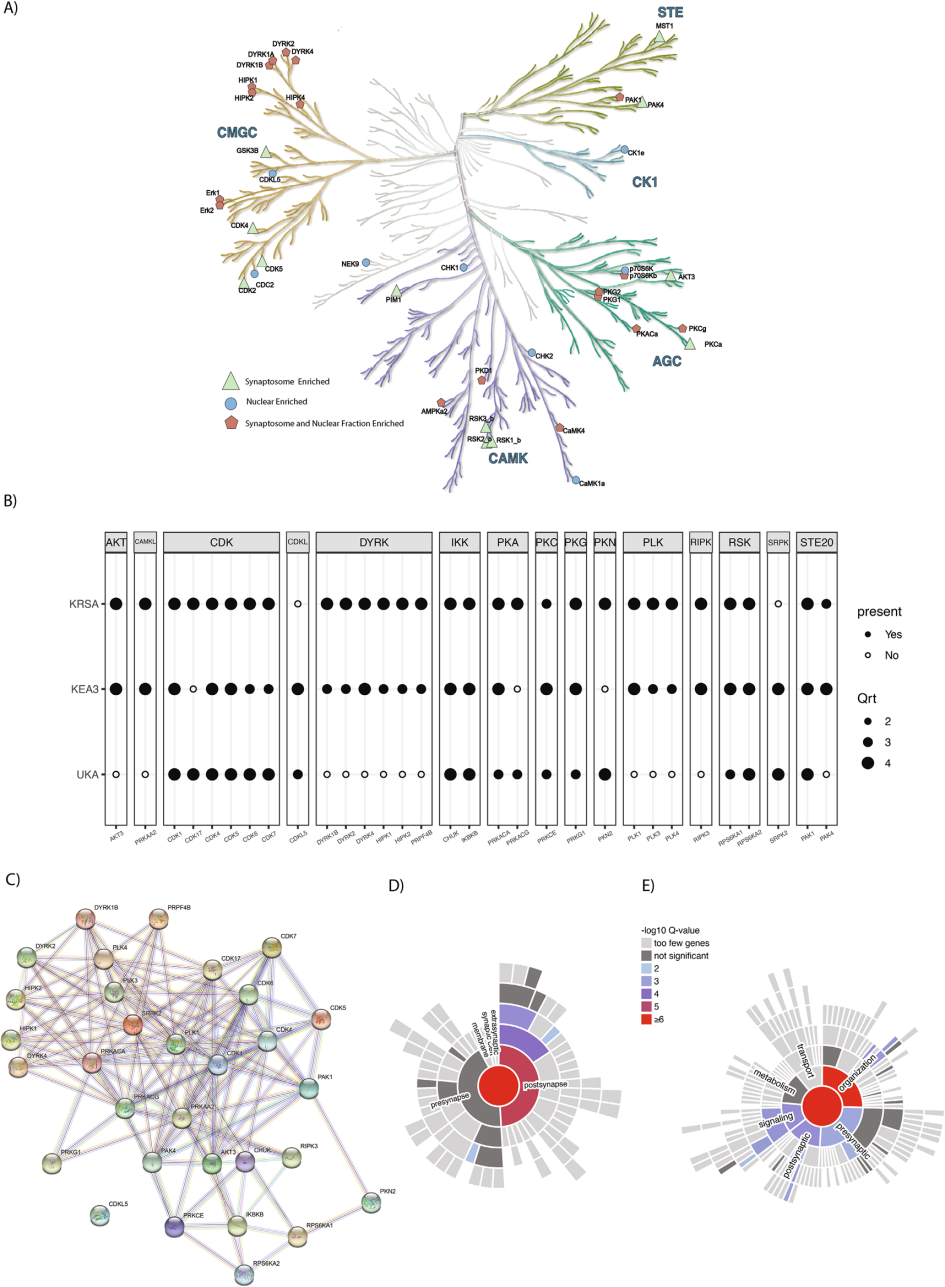
Upstream Kinase Analysis Shows a Subset of Kinases That are Actively Enriched in the Synaptosome. Using three software packages to perform upstream kinase analyses (KRSA, KEA3, and UKA), we harmonized the outputs from each tool with percentile ranking and a centralized kinome mapping reference. Criteria to select top kinases: kinase must be included in at least two tools and the mean percentile (the mean percentile is calculated by averaging the percentile ranks across all the three tools) must be >  = 0.75. (**A**) The full kinome phylogenetic tree and highlighting the top enriched kinases in each subcellular fraction (synaptosome and nuclear). The comparisons are performed with the cytosol fraction treated as the reference sample. (**B**) Top enriched kinases when comparing the nuclear and synaptosomal fractions. For visualization, the percentile values are grouped as quartiles. (**C**) STRING DB protein–protein interaction (PPI) network of the top enriched kinases when comparing the synaptosomal versus nuclear fractions. Minimum required interaction score was set as 0.2. (**D**–**E**) The SynGo gene set enrichment analysis of the top differentially active kinase networks. The protein networks are generated by extracting top 20 associated proteins from STRING (minimum required interaction score was set as 0.5). The plots are showing the top-levels Cellular Component ontology (**D**) and Biological Process ontology (**E**) terms visualized as “sunburst” plots to represent the adjusted enrichments scores (FDR) for each parent and child term. KRSA: Kinome Random Sampling Analyzer, KEA3: Kinase Enrichment Analysis, UKA: Upstream Kinase Analysis.

We used KRSA, UKA, and KEA3 for the upstream kinase analysis and the Creedenzymatic package to harmonize the results from each tool. The output form the Creedenzymatic package is percentile ranking of kinases calculated for each tool. To select the top kinases across the different tools, a kinase must be included in at least two tools and the mean percentile must be equal or higher than 0.75 (the mean percentile is calculated by averaging the percentile ranks across all the three tools). We compared the synaptosomal to the nuclear fraction as our primary analysis. The overlap of enriched kinases was extracted to explore commonly and uniquely enriched kinases in the synaptosomal versus nuclear fraction. The top kinases were overlayed on the kinome phylogenetic tree highlighting the kinases that are enriched in both the synaptosomal and nuclear fractions and kinases that are uniquely enriched in the synaptosomal or nuclear fractions (Fig. 4A). We found 18 kinases that are common between the synaptosomal and nuclear fractions, 12 kinases that are uniquely enriched in the synaptosomal fraction, and 6 kinases that are uniquely enriched in the nuclear fraction. The synaptosomal versus nuclear fraction comparisons revealed 31 enriched kinases (Fig. 4B).

### Pathway analyses for synaptosomal versus nuclear fractions

We expanded the kinase network by extracting the top 20 associated proteins for each protein kinase “hit” resulting in a protein list that consist of 446 members (Fig. 4C, Supplementary Table 1). Using this extended list, we performed gene set enrichment analysis using the SynGO curated database. The enrichment analysis showed 7 significantly (FDR < 0.05) enriched, synapse-related Cellular Component (CC) ontology terms and 21 Biological Process (BP) terms (Fig. 4D,E and Supplementary Table 2). Most of the Cellular Component enriched terms are postsynaptic-related terms including “postsynaptic density” (FDR < 3.78e−4) and “postsynaptic specialization, intracellular component” (FDR < 7.08e−3). The Biological Process terms include several terms related to “synapse organization” (FDR < 1.27e−7) and “synaptic signaling “ (FDR < 4.19e−4).

## Discussion

Using an unbiased kinome activity profiling strategy, we assessed STK activity for nuclear, cytosolic, and synaptosomal subcellular fractions from rat cortical tissue. Bioinformatic analyses revealed overall higher STK kinase activity associated with the synaptosomal fraction, as well as a enriched lists of kinases/pathways for synaptosomal versus nuclear fractions. Due to the nature of the profiling method, increased activity may be attributable to increased kinase activity, kinase gene expression levels, or both.

As detailed in the methods section, the fractionation protocol used in this study is well-established to isolate synaptoneurosomes, a crude form of synaptosomes, without the requirement of lengthy ultracentrifugation and a relatively large amount of brain tissue input. Synaptoneurosomes are structures composed of the sealed presynaptic bouton and the attached postsynaptic density. Therefore, synaptic proteins including kinases, are enriched in this fraction compared to a total cell lysate, which has been confirmed by published studies and our experiment^20^ (Fig. 1B). Furthermore, the complete lack of nuclear markers such as Histone 3 in the synaptoneurosome fraction supports its purity. Thus, this fast synaptoneurosome fractionation method can be combined with kinome profiling to provide kinase activity information with subcellular resolution. However, we also noticed the nuclear fraction produced from this method contained some synaptic proteins, such as PSD 95 and GluR1 (Fig. 1), possibly due to contamination from synaptoneurosomes^20^. This observation raised a concern that comparing kinome activity between the synaptosomal and nuclear fractions may have underestimated their differences.

We have identified a list of candidate kinases with higher activity in the synaptosomal fraction, many of which are expected as they are closely associated with synapses, both structurally and functionally. Several kinases were uniquely enriched based on their activity in the synaptosomal fraction including PAK4, CDK5, and RSK. The p21 activated kinases (PAKs) are well-known effector proteins for the Rho GTPases Cdc42 and Rac, whose function is to promote the formation and growth of synapses and spines. More specifically, PAK4, which is uniquely enriched in the synaptosomal fraction in our dataset, is linked to neuronal development^21^. Genetic manipulation of PAK4 produces underdeveloped cortical layers and loss of neuroepithelial adherens junctions^21,22^.

For cyclin-dependent kinases (CDKs), it is somewhat surprising to note the synaptosomal association of CDK2, CDK5 and nuclear enrichment of cdc2/CDK1. While there is overlap for the phosphorylation motifs for these 3 kinases, the kinase mapping software packages we used are based on several mapping databases (KRSA, UKA, KEA3) and have identified these kinases for each fraction. Many studies support that intracellular signaling cascades initiated by CDKs, especially CDK5, regulate various synaptic functions^23–25^. However, we recognize that these data results are preliminary and require formal biochemical validation in a future study.

Additionally, our data suggests that the p90 ribosomal S6 kinase (RSK) is one of the synaptosome enriched kinases. RSK is involved in synaptic plasticity and enhancement in neuronal excitability in a recent study that showed that an RNAi knockdown of RSK leads to impaired long-term synaptic facilitation^26^.

Several unexpected kinases also emerged from this screening. For example, IKB and CHUK are involved in activating the NF-kB pathway through distinct steps operating in both the cytoplasm and the nucleus; Surprisingly, these kinases were enriched in the synaptosomal fraction but not in the nucleus. Additionally, CaMKII failed to emerge from the synaptosomal list, given that it is a kinase associated with the postsynaptic site. However, the cortical tissue we used in this study was obtained from animals at a resting or naïve state; synaptic kinases like CaMKII may only show up in response to stimulation (drugs, behavior, sensory inputs, etc.). Another possibility, as mentioned above, is that contamination from synaptoneurosomes may have masked the difference.

In conclusion, we used an unbiased, peptide-based, protein kinase activity array platform to assess the STK activity in subcellular fractions. Compared to mass spectrometry methods of kinome analysis, which require significant protein inputs and enrichment of kinases before analysis, sample preparation for peptide arrays is comparatively straightforward. Additionally, due to the low protein input requirement, peptide arrays provide the opportunity to examine cellular fractions without the necessity of pooling samples. Subcellular localization of kinases is critical for their function in various biological processes and diseases. Investigating the active kinome at a subcellular resolution is key to understand the underlying mechanisms of how kinases are trafficked and activated in normal and perturbed systems.

## Supplementary Information

Supplementary Information 1.

Supplementary Information 2.

Supplementary Information 3.

## Data Availability

raw data, processed data, and full analytic scripts are deposited on GitHub. The full repository link: https://github.com/CogDisResLab/subcellular_kinome_paper.
