# Supplementary material for: Subcellular partitioning of protein kinase activity revealed by functional kinome profiling
Source: Sci Rep. 2022 Oct 15;12:17300. doi: 10.1038/s41598-022-21026-5 (PMC9569338; doi:10.1038/s41598-022-21026-5)
Supplement: Supplementary file 1 — Supplementary Information 1. [file 41598_2022_21026_MOESM1_ESM.docx]

**Subcellular partitioning of protein kinase activity revealed by functional kinome profiling**

Lauren Points, Khaled Alganem, Ali Sajid Imami, Victoria Mathis, Justin Fortune  Creeden, Robert McCullumsmith, Li-Lian Yuan

**Supplementary Methods Omnibus for the PamGene Kinome Array**

**Overview**. The PamGene platform is a well-established, highly cited, microarray technology for multiplex kinase activity profiling [1-4].

**Hardware.** Pamstation12 and PamChip4**.** The PamGene12 Kinome Array is a peptide array-based platform that facilitates the unbiased detection of kinase activity by serine/threonine (STK) or tyrosine (PTK) kinases [1-6]. The STK and PTK PamGene12 chips have 144 and 196 reporter peptides, respectively. Phosphorylation is detected in real time. Fluorescent antibodies are applied against phosphorylated residues; fluorescent intensity is a proxy for the extent of reporter peptide phosphorylation (Supplementary Figure S3). Altered kinase activity can be directly measured. For example, phosphorylation by PKA on the STK chip is concordant with its activity in solution [3]. Each peptide chip has 4-wells, and three chips can be run at the same time. Thus, there are up to 12 samples for each “run” on the array.

**Chip Coverage.** Of the about 500 kinases in the human genome [7, 8], 245/376 (65%) Ser/Thr and 89/93 (96%) Tyr kinases can be mapped to the STK and PTK chips, respectively. The chips also map about 18/21 (86%) dual specificity knases, covering about 72% of the entire kinome. The STK chip covers similar amounts of low (52%), medium (65%), and high (65%) abundance protein kinases in neurons (based on Brainseq neuron database (https://www.brainrnaseq.org/)) and has sensitivity for detection into the picogram range (unpublished data from Pamgene) for many kinases.

**Kinome Array Protocols**

**Data Generation.** Samples are prepared according to the protocols provided by PamGene Corp (https://pamgene.com/ps12/). The catalytic activity and stability of kinases are controlled by the addition of protease and phosphatase inhibitors. Peptide phosphorylation is monitored during the incubation with assay mixture, by taking images every 5 min for 60 min at exposure lengths of 5 msec, 25 msec and 100 msec, allowing real time recording of the reaction kinetics. Various internal control tests have been performed by PamGene International to ensure the sensitivity of the assay. Chip-to-chip and run-to-run technical variation (coefficient of variability (CV)) is <9% and <15%, respectfully. To account for technical variation between runs, an internal control sample may be added to account for between run variability.

**Preliminary Data Processing.** The primary output from PamStation12 is images from the Evolve kinetic image capture software. These images are then pre-processed to quantify the activity at each peptide level using the PamGene’s BioNavigator software (<https://pamgene.com/wp-content/uploads/2020/09/BioNavigator-User-Manual-vs2.3-2020.pdf>). Before proceeding to activity analysis, all peptides that appear as inactive (Raw Signal <= 5) are removed from the analysis. The dynamic range of the raw signal intensities is typically 0 - 3,000. Linear regression slope of the signal intensity as function of exposure time is used to represent the peptide phosphorylation intensity for downstream comparative analyses, averaged across the biological replicates. This is done to increase the dynamic range of the measurements. The signal ratio between case and control samples is used to calculate fold change (FC) values. Peptides with a fold change of at least 15% (ie FC > 1.15 or FC < 0.85) are considered differentially phosphorylated for the purposes of using KRSA. This threshold was chosen based on previous reports that suggest small changes in kinase activity are sufficient to trigger biologically relevant changes [5, 6, 9]. Peptides that had very low signal or an R^2^ of less than 0.90 during the corresponding linear regression are considered undetectable or non-linear in the post-wash phase and were excluded from subsequent analyses.

**Assessment of Upstream Kinases.** Peptides spotted on the array (and in general) may be phosphorylated by more than one kinase, and in many cases several different kinases. The use of two different types of chips, one for Ser/Thr kinases (STK) and one for Tyr kinases (STK) provides a starting point for assignment of kinases. There are 4 different software packages that may be deployed for assignment of upstream kinases. All of them rely, to varying extents, on publicly available mapping databases. Each has strengths and weaknesses, some of which are discussed below.

**Upstream Kinase Analysis (UKA).** This package was developed by the Pamgene Corp (s’-Hertengobosch, Netherlands)(add Table 2, URLs of packages). UKA is integrated into the manufacturer’s BioNavigator software and their recommended method. This method relies on a curated database of kinase substrate interactions created by the PamGene Corp. It takes the raw output from the PamStation as input. It then filters low intensity peptides and scales the entire dataset to the range of 0-100. It then calculates a “kinase Score” for each kinase and reports the ones with the highest score. Advantages include 1) providing results for specific kinases (as opposed to families) and 2) a low false positive rate compared to other packages. One putative weakness is that it may be too stringent for discovery-based experiments.

**Kinome Random Sampling Analyzer (KRSA).** The package was developed by the Cognitive Disorders Research Laboratory (CDRL) at the College of Medicine and Life Sciences (COMLS) University of Toledo, led by Dr. Robert McCullumsmith. The data generated from the kinome array experiment and the mapping of the PamChip file are used as input to the algorithm. Once selected, peptides are filtered out using advancement criteria, including the signal intensity at maximum exposure time and the R2 values of the linear regression of signal intensity as a function of exposure time. At the end of this step, a list of filtered peptides moves forward to the next step of the analysis.

Curation of the database of upstream kinases. KRSA relies on a curated database of upstream kinases for the peptides present on the array. Protein kinases predicted to act on phosphorylation sites within the array peptide sequences were identified using GPS 3.0 and Kinexus Phosphonet (Kinexus Bioinformatics) [10-12]. These programs provide predictions for serine-threonine kinases targeting peptide sequences ordered by likelihood of binding. The union of the highest ranked 5 kinases in Kinexus and kinases with scores more than two times the prediction threshold in GPS 3.0 were considered predicted kinases for each peptide and used in KRSA analysis [3]. This list was combined with kinases shown in the literature to act on the phosphorylation sites of the peptides via PhosphoELM (<http://phospho.elm.eu.org> ) and PhosphoSite Plus (<https://www.phosphosite.org>).

*Empirical Measures of Statistical Significance*. KRSA performs a permutation analysis (aka a Monte Carlo simulation) by taking a random sample of the same number of peptides (observed peptides) that passed the advancement criteria. For each simulation, upstream kinase counts are accumulated providing a standard normal distribution for the number of times a kinase would be predicted to be assigned by chance alone. The simulation is run 2000 times, yielding a distribution histogram, median, mean, and standard deviation values for specific kinases. A comparison is then made between the observed (ie the number of times a kinase mapped to the peptides from the actual experiment that passed QC and fold-change thresholds) and the expected (from the permutation analysis). Observed kinases are assigned a z-score value, generated based on the number of standard deviations from the mean of the expected upstream kinases. A positive z-score suggests overinvolvement (not higher activity) of this kinase in the substrate studied versus the control group. Since the z-score serves as the signifier of statistical significance (ie outside of a predetermined confidence interval), we consider any kinase with a z-score >2 to be a “hit,” warranting further assessment. In some cases, we adjust the z-score threshold (lowering the stringency) to increase the number of candidates for discovery-based studies.

*Presentation of the data in KRSA*.

Heatmaps:

The heatmaps as shown in the figure below are generated from the signal intensity data. The selection of peptides is based on the quality control criteria explained above. The values on the heatmap are the slopes of the linear models of signal intensity as a function of the exposure time.

Violin Plots:

The violin plot showcases the distribution of the signal intensity of significant peptides on a per-group basis.

Waterfall Plots:

The waterfall plots are generated from the Z score values for each kinase. These values are generated on a chip-by-chip basis and then averaged across the three. The plot shows the distribution of these three points and a red dot showing the mean z score value.

**KRSA strengths and weaknesses**. The KRSA packages was developed [13] to complement the Pamgene Corp’s UKA package. KRSA has a less stringent strategy to assign upstream kinases, providing a more putative “hits” for discovery-based studies, where findings will be confirmed with additional studies. One weakness of KRSA is that it only provides kinase families, as opposed to specific kinases. We often combine KRSA and UKA (in this order) to determine a hit “family” then identify the specific family members with UKA or another package (such as KEA3 below).

**Kinase Enrichment Analysis 3 (KEA3).** KEA3 is an upstream kinase assignment method developed by the Maayan laboratory (https://maayanlab.cloud/kea3/) that relies on the known kinase protein interactions and kinase substrate interaction data and associated co-expression and co-occurrence data [14]. The KEA3 web server takes a set of phosphorylated proteins and their fold-change value as input and returns the putative upstream kinases using the database and looking for statistically significant over representation of kinases.

**PTM Signature Enrichment Analysis (PTM-SEA).**

PTM-SEA[15, 16] is an application developed by the Broad Institute to identify putative upstream kinases. PTM-SEA is a modified form of the single sample Gene Set Enrichment Analysis (ssGSEA) with the underlying database built on top of PTMSigDB [15]. The software runs on the R Programming language. It takes in files in the Gene Cluster Text format which has the peptides and log fold change values in a specified format. The output from PTM-SEA is a list of putative upstream kinases that can phosphorylate each site.

**Integration of Upstream Kinase Assignments Across Packages.** All the tools above utilize different methods to assign upstream kinases. This necessitates the use of an integration system to identify consensus upstream kinases across datasets. For this purpose, we utilize the software Creedenzymatic [17]. The Creedenzymatic analysis takes in the results from at least two of the 4 analyses mentioned above and then generates a consensus figure with kinases deconvolved and ranked based on their presence in the results.

**Supplementary Methods References**

1. Arsenault, R., P. Griebel, and S. Napper, *Peptide arrays for kinome analysis: new opportunities and remaining challenges.* Proteomics, 2011. **11**(24): p. 4595-609.

2. Baharani, A., et al., *Technological advances for interrogating the human kinome.* Biochemical Society Transactions, 2017. **45**(1): p. 65-77.

3. Bentea, E., et al., *Kinase network dysregulation in a human induced pluripotent stem cell model of DISC1 schizophrenia.* Mol Omics, 2019. **15**(3): p. 173-188.

4. Hilhorst, R., et al., *Peptide microarrays for detailed, high-throughput substrate identification, kinetic characterization, and inhibition studies on protein kinase A.* Analytical Biochemistry, 2009. **387**(2): p. 150-161.

5. McGuire, J.L., et al., *Abnormalities of signal transduction networks in chronic schizophrenia.* NPJ Schizophr, 2017. **3**(1): p. 30.

6. Dorsett, C.R., et al., *Traumatic Brain Injury Induces Alterations in Cortical Glutamate Uptake without a Reduction in Glutamate Transporter-1 Protein Expression.* J Neurotrauma, 2017. **34**(1): p. 220-234.

7. Manning, G., et al., *The protein kinase complement of the human genome.* Science, 2002. **298**(5600): p. 1912-1934.

8. Manning, G., *Genomic overview of protein kinases.* WormBook : the online review of C. elegans biology, 2005: p. 1-19.

9. Appuhamy, J.A., et al., *Effects of AMP-activated protein kinase (AMPK) signaling and essential amino acids on mammalian target of rapamycin (mTOR) signaling and protein synthesis rates in mammary cells.* J Dairy Sci, 2014. **97**(1): p. 419-29.

10. Xue, Y., et al., *GPS 2.1: enhanced prediction of kinase-specific phosphorylation sites with an algorithm of motif length selection.* Protein Engineering Design and Selection, 2011. **24**(3): p. 255-260.

11. Wang, C., et al., *GPS 5.0: An Update on the Prediction of Kinase-specific Phosphorylation Sites in Proteins.* Genomics, Proteomics & Bioinformatics, 2020. **18**(1): p. 72-80.

12. Xue, Y., et al., *GPS: a comprehensive www server for phosphorylation sites prediction.* Nucleic Acids Research, 2005. **33**(suppl_2): p. W184-W187.

13. DePasquale, E.A.K., et al., *KRSA: An R package and R Shiny web application for an end-to-end upstream kinase analysis of kinome array data.* PLOS ONE, 2021. **16**(12): p. e0260440.

14. Kuleshov, M.V., et al., *KEA3: improved kinase enrichment analysis via data integration.* Nucleic Acids Res, 2021. **49**(W1): p. W304-W316.

15. Krug, K., et al., *A Curated Resource for Phosphosite-specific Signature Analysis.* Mol Cell Proteomics, 2019. **18**(3): p. 576-593.

16. Cox, J. and M. Mann, *MaxQuant enables high peptide identification rates, individualized p.p.b.-range mass accuracies and proteome-wide protein quantification.* Nat Biotechnol, 2008. **26**(12): p. 1367-72.

17. Khaled, A.S. Imami, and J. Creeden, *CogDisResLab/creedenzymatic: v 5.0.0 Version Reset*. 2022, Zenodo.

**Supplementary Figure 1**

**
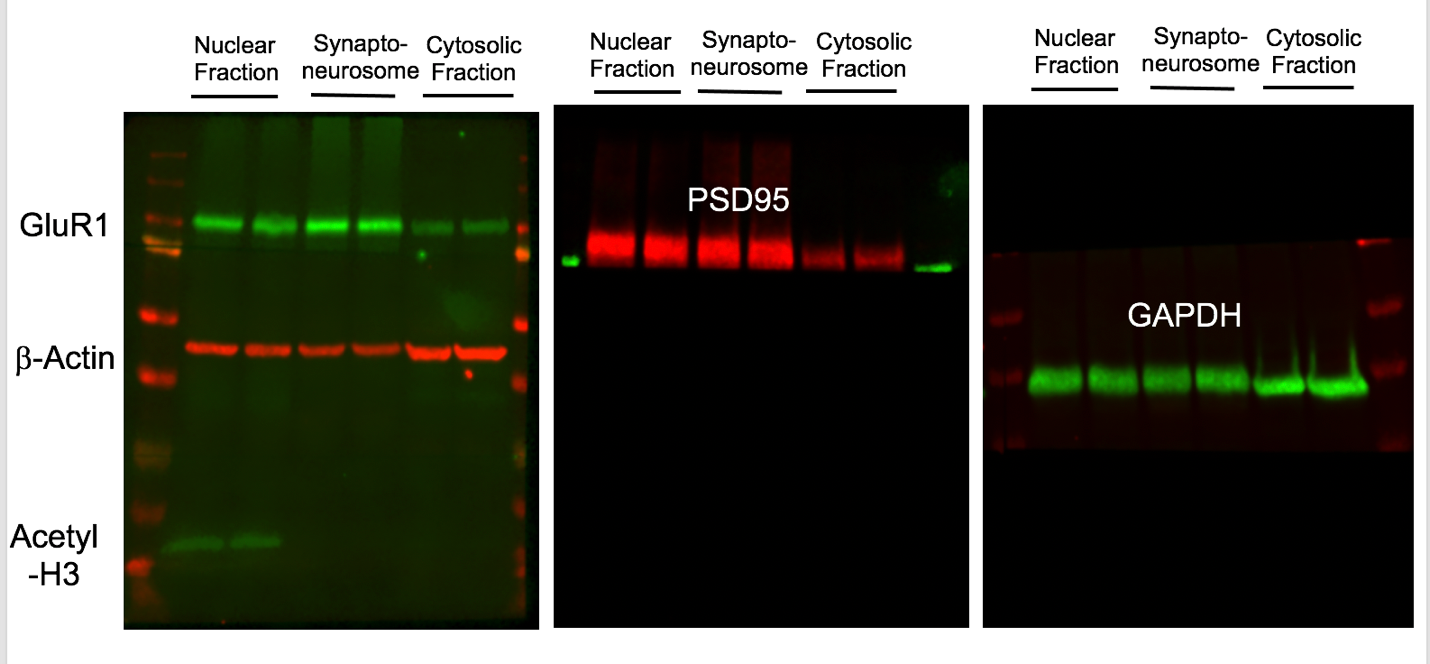
**

**Figure S1.** Original full-length image of the western blot membrane. Five proteins (GluR1, PSD95, Acetyl-3, β-actin, and GAPDH) were examined and compared among three cellular fractions (nuclear, synaptoneurosome, and cytosolic fraction).

**Supplementary Figure 2**


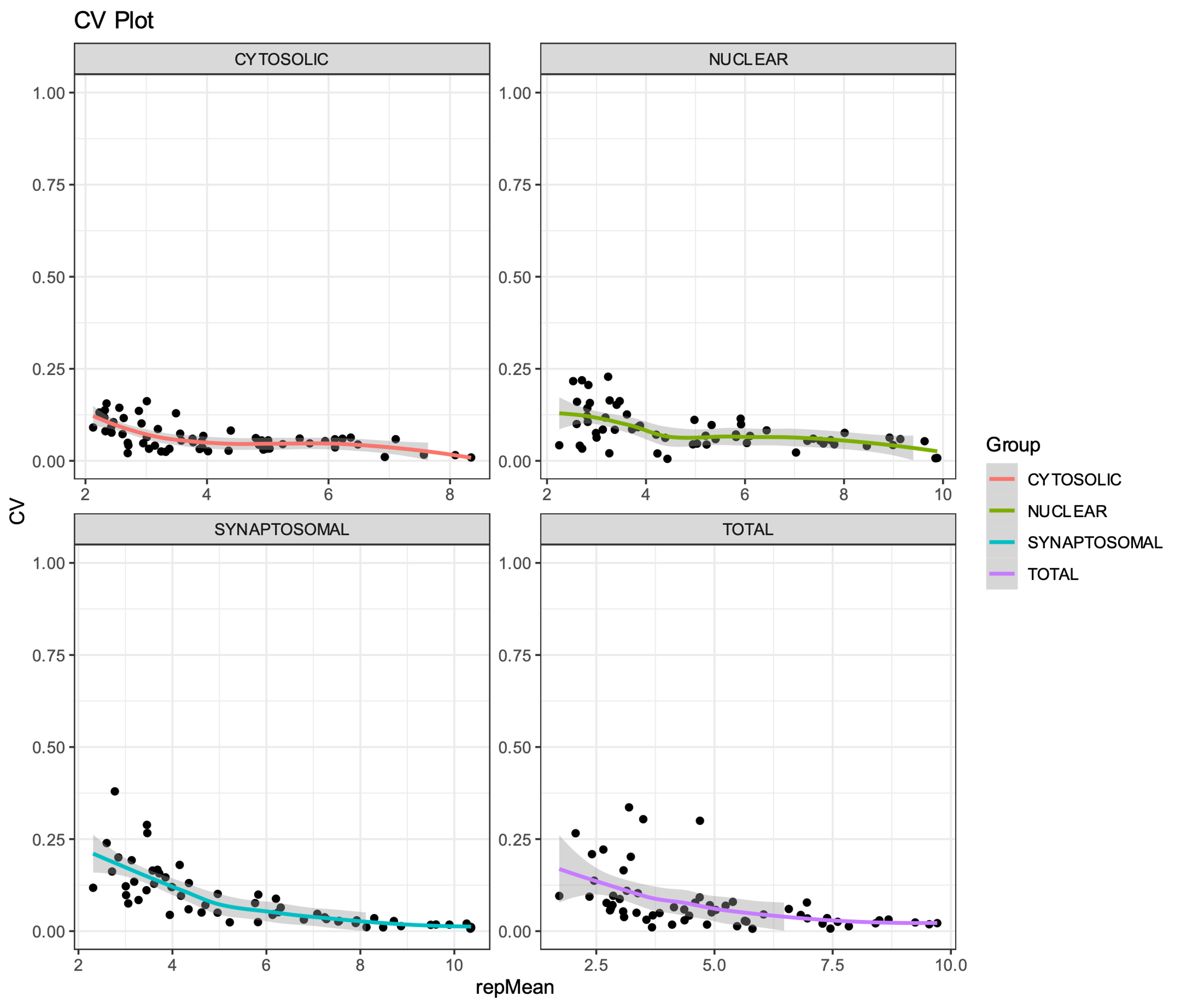


**Figure S2.** A plot of per-peptide mean values plotted against the coefficient of variation for that peptide across chips and samples. The plot is separated for each fraction and total (unfractionated brain homogenate). The median values of coefficients of variation for cytosolic (CV = 4.67%), nuclear (5.69%), and synaptosomel (3.91% ) fractions, as well as the total (4.55%) are below the manufacturer’s standard CV threshold (<9%). This indicates an acceptable level of between-sample and between-chip variability.


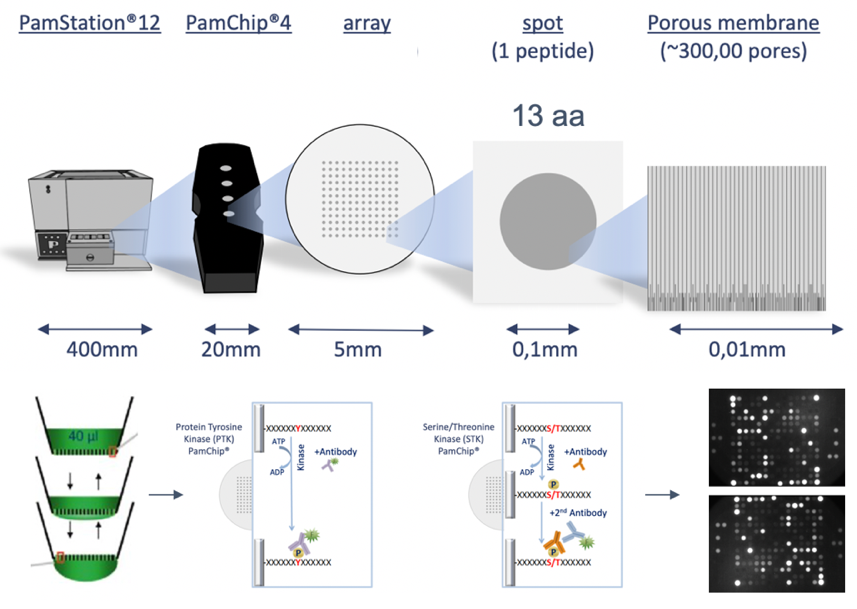
**Supplementary Figure 3**

**Figure S3: PamGene Platform Workflow**. The arrays are spotted with reporter peptides, including controls, coupled to an activated aluminum oxide surface to create a 3-D structure facilitating interactions. During an experiment, the array is incubated with lysates of cells or tissue. The active kinases in the sample will phosphorylate their target on the array. Generic fluorescent labeled antibodies that recognize phosphorylated residues are used to visualize the phosphorylation in real time. This figure was reprinted with permission from PamGene International B.V..

**Supplementary Table 1:** The list of protein obtained from the STRING DB protein-protein interaction (PPI) network of the top enriched kinases when comparing the nuclear and synaptosomal fractions. Minimum required interaction score was set as 0.2

**Supplementary Table 2:** The SynGo gene set enrichment analysis of the top differentially active kinase networks. It shows the enriched terms from the Cellular Component (CC) ontology and Biological Process (BP) ontology annotated in the SynGO database.
